# The influence of target layout and target graphic type on searching performance based on eye-tracking technology

**DOI:** 10.3389/fpsyg.2023.1052488

**Published:** 2023-02-09

**Authors:** Yaxue Zuo, Jin Qi, Zhijun Fan, Zhenya Wang, Huiyun Xu, Shurui Wang, Nieqiang Zhang, Jie Hu

**Affiliations:** ^1^School of Design, Shanghai Jiao Tong University, Shanghai, China; ^2^School of Mechanical Engineering, Shanghai Jiao Tong University, Shanghai, China; ^3^School of Mechanical Engineering, Shandong University, Jinan, China

**Keywords:** eye-tracking technology, target layout, target graphic type, searching performance, human-computer interaction

## Abstract

With the development of various intelligent technologies, the application of interactive interfaces is becoming more and more widespread, and the related researches conducted for interactive interfaces are also increasing. The purpose of this study was to explore the influence of icon layout location, icon graphic type, and icon layout method on users’ searching performance in interactive interfaces through eye-tracking technology. Participants were asked to perform search tasks for the search target (facet icon or linear icon) on each image. Thus, each trial consisted of a search task on a given image. In total, each participant had 36 trials to complete. Searching time, fixation duration, and fixation count were collected to evaluate the searching performance of participants. Results showed that when faced with familiar icons, whether the graphic type of icons was facet or linear did not affect the user’s experience, but when other factors of the interaction interface changed, facet icons provided a more stable experience for users. And compared to the rectangular layout, the circular layout method provided a more stable experience for users when the location of icons in the interactive interface changed, but icons located in the top half of the interactive interface were easier to find than those located in the bottom half, regardless of whether the layout was circular or rectangular. These results could be used in the layout and icon design of the interactive interfaces to facilitate their optimization.

## 1. Introduction

Digital interactive interfaces are widely used in driving environment (Schmidt et al., 2010; Kataria et al., 2015; Costa et al., 2017; Wang et al., 2021; He et al., 2022), industrial production environment (Gamecho et al., 2015; Diego-Mas et al., 2017), medical diagnosis environment (Erol Barkana and Açık, 2014; Açık et al., 2016; Meena et al., 2018), leisure and entertainment environment (Lu and Jia, 2014; Kanaan and Moacdieh, 2021), etc. Especially with the development of intelligent technology, many physical interactive interfaces have been gradually transformed into digital interactive interfaces (Shneiderman, 1997), which, together with touch screen (Jansen and Dragicevic, 2013), eye-movement control technology (Meena et al., 2018; Bozomitu et al., 2019), electroencephalographic (EEG) control technology (Vourvopoulos et al., 2019; Si-Mohammed et al., 2020), voice control technology (Hogan et al., 2017), motion capture technology (Kirsh, 2013), action recognition technology (Alaoui et al., 2015), etc., can achieve good user experiences and effectively enhance the convenience, intelligence and adjustability of interactive interfaces (Rogers et al., 2012).

There are a number of studies that have been conducted on interactive interfaces. Some researchers have carried out researches on the evaluation of interfaces (Purchase et al., 2011; Deng et al., 2020). For example, Zen and Vanderdonckt (2014) studied the esthetics of graphical user interface (GUI) and put forward a methodology for the evaluation of GUIs esthetics. Oulasvirta et al. (2018) also conducted researches on GUI evaluation, and based on these researches, they developed Aalto Interface Metrics (AIM), a GUI evaluation tool that contains several validated models and metrics of user perception and attention into an easy-to-use online service for the evaluation of GUI designs. Uribe et al. (2017) also developed an evaluation model, which was mainly applied to website interfaces. Unlike the interface evaluation models proposed by other researchers, their model was obtained by applying a stepwise regression algorithm, and inferred from the user’s first impression by analyzing three different visual characteristics of website screenshots: texture, luminance, and color.

The design and optimization of interactive interfaces is also a hot topic in the field of interactive interface research (Liang et al., 2010; Tao et al., 2018; Bunian et al., 2021). Shneiderman (1997) had systematically studied human-computer interaction as early as 25 years ago, and proposed a series of strategies for the user interface design. Zhou and Wei (2010) clarified the human-oriented theory of interface design and applied this theory in the whole process of interface design. Rice and Alm (2008) took the older people as the focus when conducting research on interface design. They explored a range of methodologies and interactive approaches designed to support older people who have difficulties in using current interface models for digital television (DTV). May et al. (2005) also focused on the elderly in their interface research. They conducted an experiment investigating the effects of providing landmarks within the instructions presented by an in-vehicle navigation system, found significant differences between older and younger drivers, and further discussed the implications of the results for the design of in-car interfaces for the older drivers. Sousa et al. (2008) and Han et al. (2020) considered business processes in the design of user interfaces, and used the model-driven method to autonomously derive user interfaces that conform to business processes.

However, the studies on user interfaces mentioned above, no matter the evaluation or the design and optimization of the interfaces, started from the macroscopic method level, and did not focus on the layout of the user interfaces and the influence of the layout of the interfaces on the user experience. In fact, regarding the layout of interfaces, some researchers have also conducted corresponding researches in recent years (Joshi and Shukla, 2017; Chung et al., 2019; Jiang et al., 2019; Todi et al., 2019; Manandhar et al., 2020; Swearngin et al., 2020; Li et al., 2021). Poorly designed workstations would cause or complicate many musculoskeletal disorders (MSDs), such as carpal tunnel syndrome and tendonitis. In order to resolve this problem, Holman et al. (2003) proposed to use linear programming to optimize control panel layout design to minimize the reach and movement distances required by an operator, and in their study, the reconfiguring of the control panel layout effectively reduced the ineffective movement of operators. Erol Barkana and Açık (2014) used eye-movement data in conjunction with task completion time and accuracy to evaluate whether a set of changes in a surgical interface (SI) layout could improve the user experience. In their work, they redesigned the SI with considering the factors of usability and functionality during the process, and the configurations of SI that had been improved mainly comprised various combinations of menu-based command controls, visual display of multi-modal medical images, 2D and 3D models of the surgical environment, graphical or tabulated information, visual alerts, etc. Diego-Mas et al. (2019) used eye-tracking, mouse movements, and genetic algorithms to optimize the layout of user interfaces. In their work, the mouse and eye-tracking data were gathered by monitoring the operators who were using the control panel to develop several predefined tasks, and the optimal layout was obtained by using a genetic algorithm. Wang et al. (2020) used eye-control technique to investigate the influence of target layout and target picking method on picking time and dragging performance. Kanaan and Moacdieh (2021) studied whether the differences in the eye movement led by the display clutter in the website layout could be detected in the first few seconds of a search task using a realistic display, both with or without time pressure. In the above-mentioned studies on the interface layouts, the researchers mainly considered the layout distribution of larger modules in the interactive interfaces, and also paid attention to the location of target elements in the interface layouts; however, they did not consider the specific distribution of individual elements in individual modules; nor did they further investigate how the target element location factors and the way they were arranged would affect the experimental results; nor did they consider the influence of the target graphic types on the experimental results.

This study focused on whether the graphic types, layout methods, and layout locations of target icons in the interactive interface would affect the user experience of the interactive interface. In this study, the automobile console interface was used as the research carrier; eye-tracking technology was used to collect experimental data (Goldberg and Kotval, 1999), and searching time, fixation duration, and fixation count were used as the quantitative metrics of the searching performance (Moacdieh and Sarter, 2015; Moacdieh and Sarter, 2017a; Moacdieh and Sarter, 2017b), that is, these quantitative metrics were used as the criteria for judging the effectiveness of the interactive interface. Through this study, we concluded that different layout methods of the target icons would affect the user experience of the interface, and the degree of the influence of the target icons’ locations and graphic types on the user experience of the interface was greatly related to the layout methods of the target icons.

## 2. Materials and methods

### 2.1. Participants

Participants were 39 students (15 women and 24 men) from the Shanghai Jiao Tong University (SJTU; average age: 24.2 ± 0.9 years). All participants had not been familiar with the experiment in advance, and all participants had self-reported normal or corrected to normal eyesight without glasses. We randomly selected one participant at a time to complete the experiment independently. The study was approved by the SJTU Institutional Review Board.

### 2.2. Experiment setup

Participants sat at a distance of 45 cm (68.2° visual angle) from a 24-inch monitor (1920 × 1,200 pixels). A Tobii Pro X2-30 eye tracker (sampling rate: 30 Hz) was attached to the monitor.

### 2.3. Stimuli

**Figure 1:**
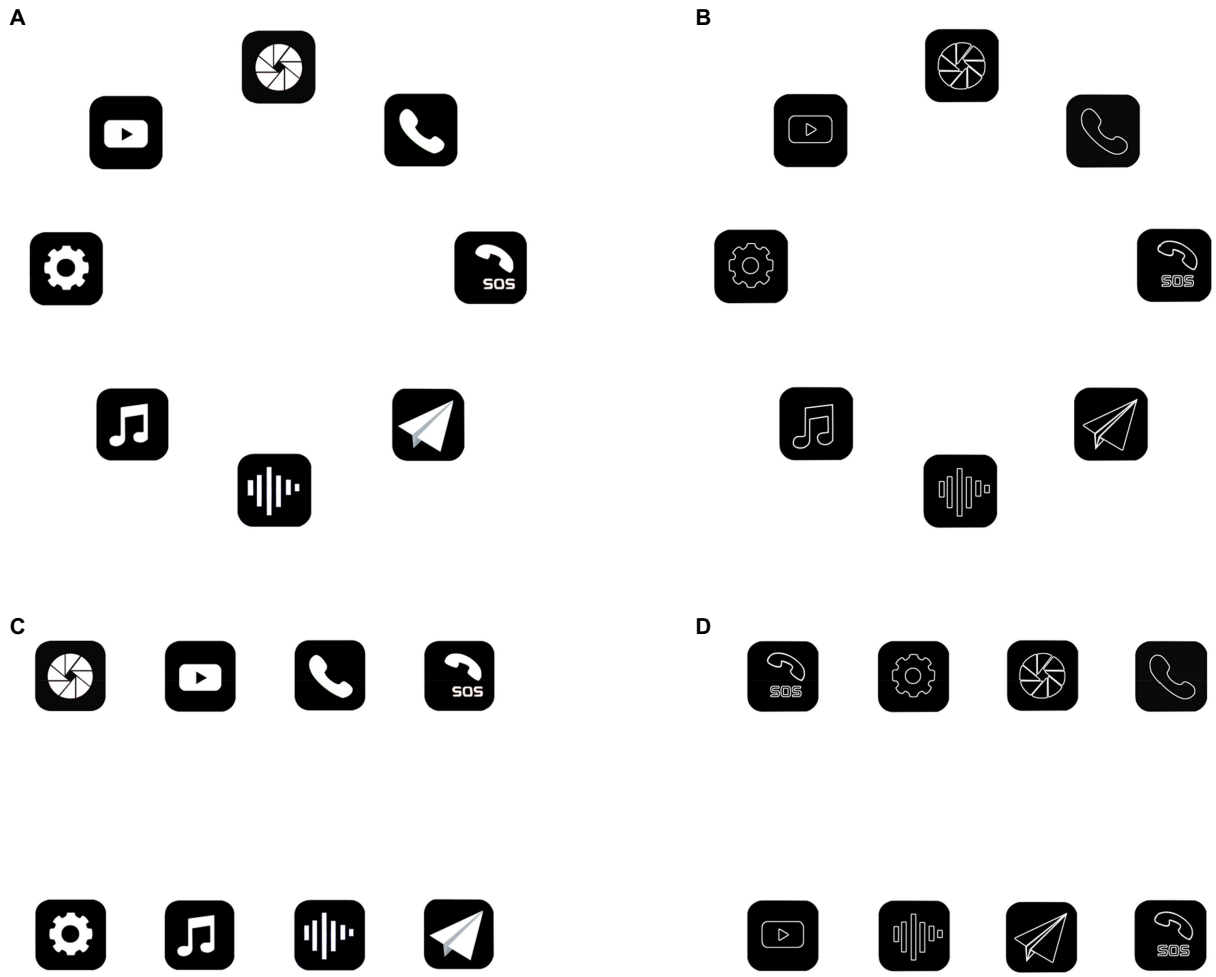
Display of icon graphic types and icon layout methods (taking Geely brand as an example). **(A)** Circular layout of facet icons. **(B)** Circular layout of linear icons. **(C)** Rectangular layout of facet icons. **(D)** Rectangular layout of linear icons.

The research stimuli consisted of 36 images of common operation icons on the automobile console interface. All of the images, when displayed, filled the whole 1920 × 1200 screen. To include a wide range of icons, the icons were selected from tree brands of cars: Jeep, Geely, and Tesla. In order to achieve the purpose of the experiment, the icons we used in the experiment are all common in life with the clear meanings. In addition, we processed these icons to ensure that each icon for each brand of car contains two graphic types (facet type, linear type) and two overall layout methods (circular layout, rectangular layout), as shown in Figure 1. All the icons in the images have the same size of 100px × 100px, and the color matching of the icon graphics themselves were white icons with black background, while the overall background of the image was uniformly white. The 8 icons of the same brand and graphic type were arranged together as one image, where the circular layout was a circle with a radius of 75 mm (the distance from the center of the circle to the center of each icon); the rectangular layout is two rows, with the center distance of 50 mm in the horizontal direction and 100 mm in the vertical direction of the adjacent icons. All images were stored as bitmaps in Jpeg format.

### 2.4. Search target

**Figure 2:**
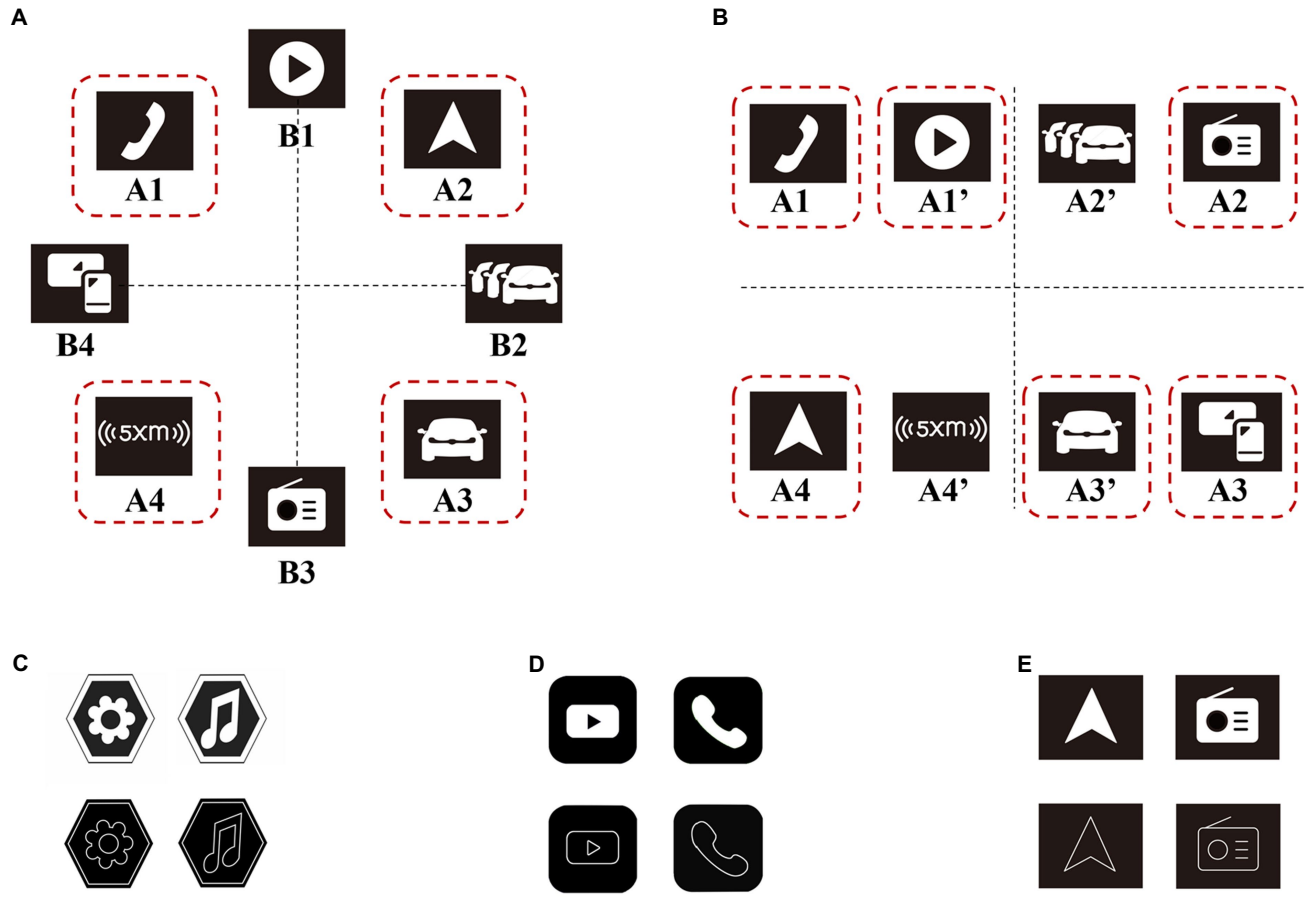
Locations of icons in different layout methods (taking Tesla brand as an example), and target search icons for different brands. **(A)** Locations of icons in circular layout, and the locations in the red dashed box are the optional locations of the target icons. **(B)** Locations of icons in rectangular layout, and the locations in the red dashed box are the optional locations of the target icons. Note: The dashed lines and dashed boxes did not appear in the experimental stimuli, and appeared in this figure only to facilitate the readers’ understanding. **(C)** Two target search icons in Jeep: “Setting”, “Music”. **(D)** Two target search icons in Geely: “Video”, “Phone”. **(E)** Two target search icons in Tesla: “Navigation”, “Radio”.

Half of the images were assigned facet icon search targets and half were assigned linear icon search targets. Icons in 24 images showed a circular layout and icons in 12 images showed a rectangular layout, with 8 locations available for icons placement in both layouts, as shown in Figures 2A, B.

To eliminate the effect of repeated search on the experimental results, we set the target search icons for each brand to be different ones: two search targets in Jeep were “Setting” and “Music”; two search targets in Geely were “Video” and “Phone”; and two search targets in Tesla were “Navigation”, and “Radio”, as shown in Figures 2C–E.

### 2.5. Experiment design

The independent variables in this study were target graphic type (facet type, linear type) and target location (A1, A2, A3, A4 in circular layout; A1, A1’, A2, A3, A3’, A4 in rectangular layout) which were both manipulated within-subjects. Layout method (circular layout or rectangular layout) was treated as a blocking variable given that we were not interested in comparing circular layout vs. rectangular layout; rather, the focus was on the target graphic type and the target location.

Participants were asked to perform search tasks for the search target (facet icon or linear icon) on each image. Thus, each trial consisted of a search task on a given image. In total, each participant had 36 trials to complete. The images were ordered so that participants first did 24 trials with the circular layout images, and then after a 5-min break, they did 12 trials with the rectangular layout images. During the experiment, each time the participants completed a trial, a gray blank screen would appear for 2 s, and then the participants would enter the next trial. In each trial, an instruction page containing textual instruction would first appear for 3 s on the computer screen to tell the participants what target icon they needed to search for in this trial, and then, the image with icons would appear. At this time, the participants started searching for the target icon, and there was no time limit for the search process until the participants completed the search. The images in each set of trials were randomly ordered, ensuring only that the searching times of the facet target icons are equal to those of the linear target icons in each set of trials.

### 2.6. Dependent variables

The dependent variables were searching time, fixation duration, and fixation count, which together constituted the participants’ searching performance. Searching time was calculated from when the image with icons appeared until participants clicked on the location of the target icon. Fixation duration was the time that the eyes remain on one point or one area in each image during the search process (Geisen and Romano Bergstrom, 2017). Generally, the average fixation duration is approximately 250–300 ms (Irwin, 2001). In this study, fixation duration referred to the total fixation duration of the eyes on each image during the search process; Fixation count was the total number of fixations on each image during the search process (Geisen and Romano Bergstrom, 2017). Any trial that contained an error was not included in calculation of searching time, fixation duration, and fixation count. Two types of errors were considered: miss errors (wrong target) and giving-up errors.

### 2.7. Experiment procedure

Participants were asked to complete a profile questionnaire (demographic information) and an informed consent form first. Then, experiment leader explained the related matters and procedure of the experiment, and participants completed five training tasks. Then, the formal experiment started. First, participants were told that they were about to enter the formal trials, which were divided into two sets, each with no time limit and a 5-min break between the two sets. Next, the eye tracker was calibrated using a nine-point grid. Then, an image with facet icons or linear icons would appear after a 3 s instruction page, and participants could look as long as they need. Participants pressed the right arrow key to proceed, at which point a gray blank image would appear, and after 2 s, the next image with icons would appear after a 3 s instruction page until participants finish the set of trials. For each trial, participants had to click on the assumed location of the target icon with the left mouse key, or right-click anywhere to give up.

### 2.8. Data analysis

In this study, Wilcoxon test and Friedman test were used to analyze the results of circular layout and rectangular layout, because according to the Shapiro–Wilk test, some data in both layouts significantly deviated from the normal distribution.

## 3. Results

### 3.1. Circular layout

#### 3.1.1. Searching time

**Figure 3:**
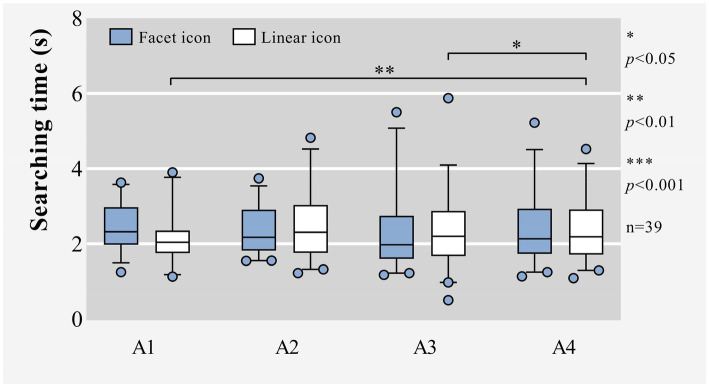
Box plots of raw data for the searching time in circular layout.

For searching time, there was no significant effect of icon graphic types, as shown in Table 1 and Figure 3. Under the premise of controlling the variable of location, the different graphic types of icons would not affect the time for participants to search for the target icons. Similarly, when the target was a facet icon, there was also no significant effect of target locations on searching time, with *p* = 0.360 greater than 0.05 in Friedman test. However, when the target was a linear icon, there was a significant effect of target locations on searching time, with *p* = 0.013 less than 0.05 in Friedman test. According to Table 2, when the target icon was linear type and located at A4, participants spent more time searching for it than that at A1 or A3, and there was no significant difference in the searching time spent by participants when the target icon was located at A1 or A3.

**Table 1:** Results of the searching time along the different locations.

Target location	Facet icon, median (SD)	Linear icon, median (SD)	Wilcoxon ranked sign test
A1	2.412 (0.604)	2.201 (0.733)	Z = 1.988 (*p* = 0.058)
A2	2.348 (0.585)	2.480 (0.850)	Z = −0.712 (*p* = 0.477)
A3	2.342 (1.026)	2.294 (1.019)	Z = 1.291 (*p* = 0.197)
A4	2.431 (0.916)	2.347 (0.797)	Z = 0.841 (*p* = 0.400)

**Table 2:** Wilcoxon ranked sign test for the searching time when the target icon is linear type.

	A2 − A1	A3 − A1	A4 − A1	A3 − A2	A4 − A2	A4 − A3
Z	1.518	0.673	**2.343**	− 0.960	1.478	**2.055**
*p*	0.129	0.501	**0.019**	0.337	0.139	**0.040**

The bolded font indicates that the *p*-values for these columns are all less than 0.05.

#### 3.1.2. Fixation duration

For fixation duration, there was no significant effect of icon graphic types, as shown in Table 3. Under the premise of controlling the variable of location, the different graphic types of icons would not affect the fixation duration in the process of searching for the target icons for participants. Similarly, when the target was a facet icon, there was also no significant effect of target locations on fixation duration in the searching process, with *p* = 0.463 greater than 0.05 in Friedman test. However, when the target was a linear icon, there was a significant effect of target locations on fixation duration in the searching process, with *p* = 0.002 less than 0.05 in Friedman test. According to Table 4, when the target icon was linear type and located at A2, the fixation duration of participants in the process of searching for it was significantly longer than that at A1, A3, or A4, and there was no significant difference in the fixation duration, when the target icon was located at A1, A3 or A4.

**Table 3:** Results of the fixation duration along the different locations.

Target location	Facet icon, median (SD)	Linear icon, median (SD)	Wilcoxon ranked sign test
A1	1.989 (0.579)	2.006 (0.770)	Z = −0.530 (*p* = 0.596)
A2	2.066 (0.946)	2.114 (0.930)	Z = −1.102 (*p* = 0.270)
A3	1.824 (0.706)	1.989 (0.849)	Z = −1.012 (*p* = 0.312)
A4	2.075 (0.830)	2.039 (0.801)	Z = 0.544 (*p* = 0.586)

**Table 4:** Wilcoxon ranked sign test for the fixation duration when the target icon is linear type.

	A2 − A1	A3 − A1	A4 − A1	A3 − A2	A4 − A2	A4 − A3
Z	**2.871**	**−** 1.089	0.893	**− 2.854**	**− 2.146**	1.891
*p*	**0.004**	0.276	0.372	**0.004**	**0.032**	0.059

The bolded font indicates that the *p*-values for these columns are all less than 0.05.

#### 3.1.3. Fixation count

For fixation count, there was no significant effect of icon graphic types, as shown in Table 5. Under the premise of controlling the variable of location, the different types of icons would not affect the fixation count in the process of searching for the target icons for participants. Similarly, when controlling the variable of target graphic type, there was no significant effect of target locations on the fixation count in the searching process. When the target was a facet icon, Friedman test for different locations yielded *p* = 0.091, greater than 0.05. When the target was a linear icon, Friedman test for different locations yielded *p* = 0.203, also greater than 0.05.

**Table 5:** Results of the fixation count along the different locations.

Target location	Facet icon, median (SD)	Linear icon, median (SD)	Wilcoxon ranked sign test
A1	3.564 (1.470)	3.111 (1.373)	Z = 1.894 (*p* = 0.058)
A2	3.043 (1.349)	3.590 (1.597)	Z = −1.495 (*p* = 0.135)
A3	3.872 (2.015)	3.359 (1.597)	Z = 1.603 (*p* = 0.109)
A4	3.744 (1.491)	3.840 (1.818)	Z = −0.276 (*p* = 0.782)

### 3.2. Rectangular layout

Based on the results of the pre-experiment (with 17 participants) before this formal experiment, we had found that the change of the icon graphic type did not bring statistical differences in either the circular layout or the rectangular layout under the premise of keeping the icon location unchanged. Therefore, we decided to analyze the effect of the graphic types only in the circular layout and not in the rectangular layout in this formal experiment, so as to reduce the total number of trials and reduce the fatigue of the participants during the experiment. This is also the reason why in the formal experimental design, we set the circular layout trial to 24 times and the rectangular layout trial to 12 times.

At the same time, according to the analysis of the circular layout results in this formal experiment, we again confirmed that for the same location, the graphic types of the target icon would not have a significant effect on the searching performance, so in the rectangular layout, we only analyzed the effect of location on the searching performance (searching time, fixation duration, and fixation count). Based on the results of the pre-experiment and the results of the circular layout in this formal experiment, we can ensure the correctness of this decision to some extent.

#### 3.2.1. Searching time

**Figure 4:**
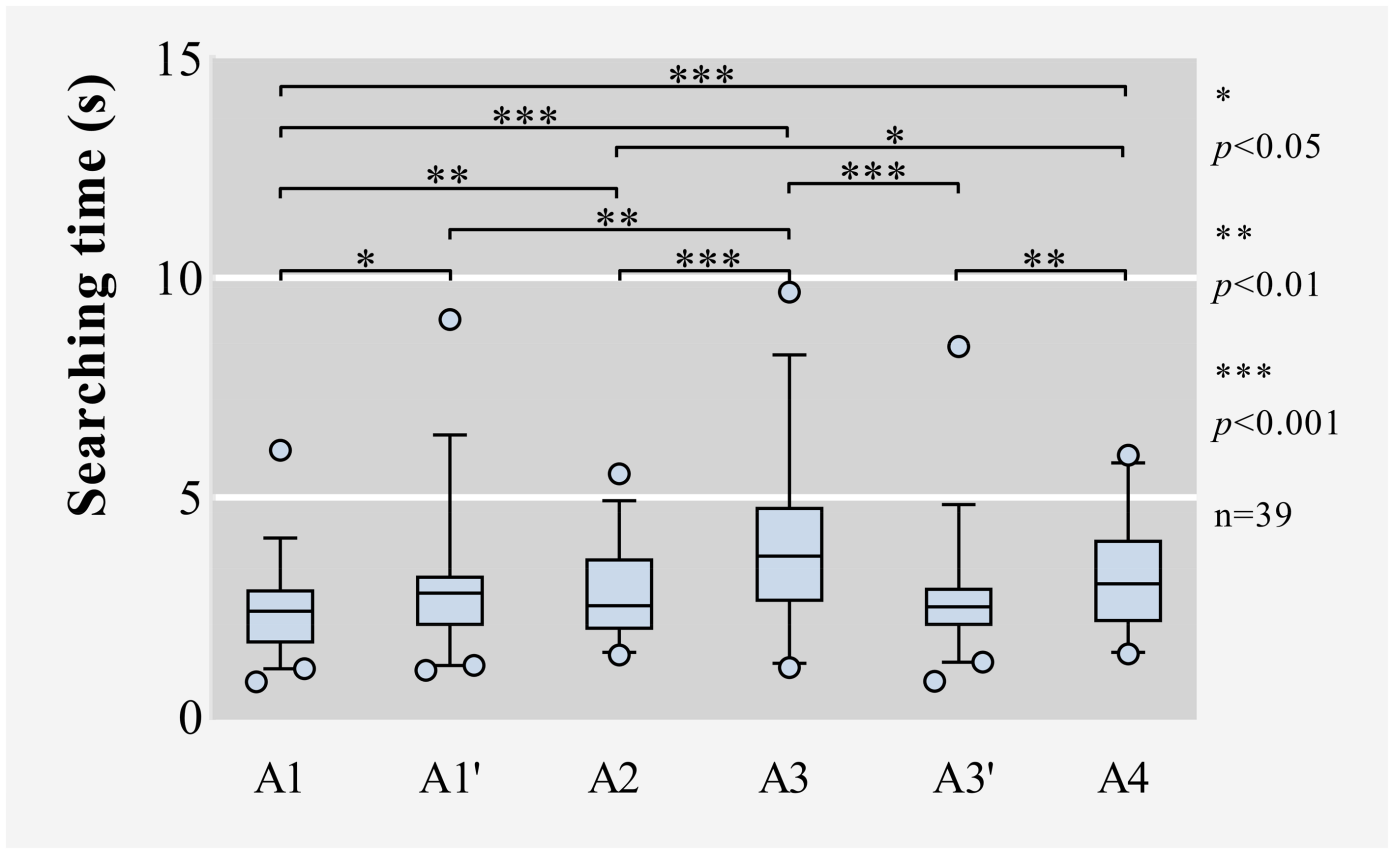
Box plots of raw data for the searching time in rectangular layout.

For searching time, there was a significant effect of icon locations, with *p* = 0.000 less than 0.05 in Friedman test, as shown in Table 6 and Figure 4. According to Table 7, when the target icon was located at A3, participants spent more time searching for it than that at A1, A1’, A2, or A3’; when the target icon was located at A4, participants spent more time searching for it than that at A1, A2, or A3’; and when the target icon was located at A1, participants spent less time searching for it than that at A1’, A2, A3, or A4.

**Table 6:** Results of the searching time along the different locations.

Target location	A1	A1’	A2	A3	A3’	A4
Median (SD)	2.423 (1.018)	2.939 (1.464)	2.865 (1.086)	3.959 (2.000)	2.751 (1.286)	3.253 (1.356)
*p* (Friedman test)	0.000					

**Table 7:** Wilcoxon ranked sign test for the searching time (only for *p* < 0.05).

	A1’ − A1	A2 − A1	A3 − A1	A4 − A1	A3 − A1’	A3 − A2	A4 − A2	A3 − A3’	A4 − A3’
Z	**2.303**	**3.154**	**4.110**	**3.489**	**3.154**	**3.489**	**2.344**	**4.496**	**2.719**
*p*	**0.021**	**0.002**	**0.000**	**0.000**	**0.002**	**0.000**	**0.019**	**0.000**	**0.007**

The bolded font indicates that the *p*-values for these columns are all less than 0.05.

#### 3.2.2. Fixation duration

For fixation duration, there was a significant effect of icon locations, with *p* = 0.000 less than 0.05 in Friedman test, as shown in Table 8. According to Table 9, when the target icon was located at A3, the fixation duration of participants in the process of searching for it was significantly longer than that at A1, A1’, A2, or A3’; when the target icon was located at A4, the fixation duration of participants in the process of searching for it was significantly longer than that at A1, A2, or A3’; and when the target icon was located at A1, the fixation duration of participants in the process of searching for it was significantly shorter than that at A1’, A2, A3, or A4.

**Table 8:** Results of the searching time along the different locations.

Target location	A1	A1’	A2	A3	A3’	A4
Median (SD)	2.134 (0.973)	2.584 (1.343)	2.435 (1.044)	3.288 (1.723)	2.435 (1.139)	2.680 (1.192)
*p* (Friedman test)	0.000					

**Table 9:** Wilcoxon ranked sign test for the fixation duration (only for *p* < 0.05).

	A1’ − A1	A2 − A1	A3 − A1	A4 − A1	A3 − A1’	A3 − A2	A4 − A2	A3 − A3’	A4 − A3’
Z	**2.387**	**2.791**	**3.677**	**3.184**	**2.840**	**3.475**	**2.132**	**4.208**	**1.996**
*p*	**0.017**	**0.005**	**0.000**	**0.001**	**0.005**	**0.001**	**0.033**	**0.000**	**0.046**

The bolded font indicates that the *p*-values for these columns are all less than 0.05.

#### 3.2.3. Fixation count

For fixation count, there was a significant effect of icon locations, with *p* = 0.002 less than 0.05 in Friedman test, as shown in Table 10. According to Table 11, when the target icon was located at A3, the fixation count of participants in the process of searching for it was greater than that at A1, A1’, A2, or A3’; when the target icon was located at A4, the fixation count of participants in the process of searching for it was greater than that at A1, A1’, A2, or A3’; and when the target icon was located at A1, the fixation count of participants in the process of searching for it was less than that at A2, A3, or A4.

**Table 10:** Results of the fixation count along the different locations.

Target location	A1	A1’	A2	A3	A3’	A4
Median (SD)	3.769 (2.108)	4.154 (2.455)	4.564 (1.463)	6.205 (4.008)	4.462 (2.501)	5.564 (2.664)
*p* (Friedman test)	0.000					

**Table 11:** Wilcoxon ranked sign test for the fixation count (only for *p* < 0.05).

	A2 − A1	A3 − A1	A4 − A1	A3 − A1’	A4 − A1’	A3 − A2	A4 − A2	A3 − A3’	A4 − A3’
Z	**2.570**	**3.726**	**3.402**	**2.823**	**2.689**	**2.627**	**2.787**	**2.818**	**2.599**
*p*	**0.010**	**0.000**	**0.001**	**0.005**	**0.007**	**0.009**	**0.005**	**0.005**	**0.009**

The bolded font indicates that the *p*-values for these columns are all less than 0.05.

### 3.3. Layout method

We did not make a direct comparison of the layout methods because it is impossible to guarantee that the target icon is in the same location in the images of two different layouts at the same time, which means that we cannot control the variable of location in the direct comparison of the two layout methods to achieve the purpose of comparison. However, by indirectly comparing the results of the analysis of circular layout and rectangular layout, we can conclude that the different layout methods have an effect on the participants’ performance in searching for the target icons. For example, in the circular layout, the effect of the variable of location on participants’ searching performance was far less than that in the rectangular layout.

## 4. Discussion

### 4.1. Target graphic type

To determine the effect of graphic type on user’s performance or user experience when looking for some icons in the interactive interfaces, we used the icons with different graphic types (facet icons, linear icons) as the search targets and compared participants’ eye-movement data for the facet target icons and linear target icons. Results indicated that there was no significant difference in the searching performance (searching time, fixation duration, and fixation count) in the process of searching for the target icons for participants between the facet and linear icons, when other variables, including location, were not changed. One possible reason could be that the icons we used in the stimuli of the experiment are all common in life with the clear meanings, so they would not cause cognitive difficulties for the participants, regardless of whether these icons are facet icons or linear icons. It implies that when the icons used in the interactive interfaces are easy to comprehend for users, the graphic types of these icons will not cause any difference in the user experience.

### 4.2. Target location

In this study, there were two layout methods (circular layout, rectangular layout), each with 8 evenly distributed positions. We set 4 layout locations for the target icons in the circular layout and 6 layout locations for the target icons in the rectangular layout. According to the results, in the circular layout, when the icons were facet icons, the different locations of the target icons did not cause significant differences in the participants’ searching performance, while when the icons were linear icons, the different locations of the target icons caused significant differences in the participants’ searching performance. Specifically, when the target was linear icon and located at A4, the searching time of participants in searching for the target icon was significantly longer than that when the target icon was located at A1 or A3; when the target was linear icon and located at A2, the fixation duration of participants in searching for the target icon was significantly longer than that when the target icon was located at A1, A3, or A4. This means that, on the whole, in the circular layout, when the icons are facet icons, the change of the locations of these icons would not have a significant effect on the user experience; when the icons are linear icons, the user experience would be significantly affected by the change of the location of the target icon. Specially, when all icons are in a circular layout, the target icon located at the bottom left would increase the user’s searching time, and the total distance of eye movement would be longer and the searching area of eye movement would be larger during the searching process than that at the top right.

However, in the rectangular layout, the different locations of the target icons caused more significant differences in the participants’ searching performance compared to the circular layout. When the target icon was located at A1, the searching time and fixation duration during the searching process were significantly less than those in A1’, A2, A3, or A4; when the target icon was located at A3, the searching time and fixation duration were significantly longer than those in A1, A1’, A2, or A3’; and when the target icon was located at A4, the searching time and fixation duration during the searching process were significantly longer than those in A1, A2, or A3’. This means that, on the whole, the user experience would be significantly affected by the location of the target icon in the rectangular layout. Specially, in the rectangular layout, when the target icon is located in the lower half, it would significantly increase the user’s searching time and fixation duration, while when the target icon is located in the upper half, it would significantly reduce the user’s searching time and fixation duration, and when the target icon is located in the upper left, it would achieve the effect of minimizing the user’s searching time and fixation duration. At the same time, when the target icon is located in the upper half, moving the target icon in parallel to approach the center of the interface would not reduce the searching time or fixation duration; however, when the target icon is located in the lower half, moving the target icon parallel to the center of the interface would effectively reduce the searching time and fixation duration, and thus increase the user’s search efficiency.

### 4.3. Target layout method

Obviously, according to the above discussion about the target graphic type and target location, it can be known that different icon layout methods would cause different effects on the participants’ searching performance. Compared with the circular layout, the change of the target icon location in the rectangular layout obviously caused more significant effects on the participants’ searching performance, which means that the circular interface layout will give users a more stable user experience when the locations of different elements in the interface change. However, it should be emphasized that more stable does not mean more efficient. It only means that changing the locations of elements in the interface in a circular layout has less impact on the users than changing the locations of elements in the interface in a rectangular layout. This may be related to people’s reading habits. In this study, all participants are Chinese and familiar with the left-to-right and top-to-bottom reading habits, and the rectangular layout of interface icons in this study was similar to the normal layout of fonts in books and other materials, so the participants naturally had a left-to-right and top-to-bottom search pattern when searching for the target icons, thus producing the relevant results in the experiments of this study. For example, the target icons were easier to be found when they were located on the top left.

However, the circular layout of the icons in this study broke the familiar layout pattern of the participants, and thus avoided the influence of the layout factors on the participants’ searching behaviors, so the participants’ searching performance in the circular layout was more stable than that in the rectangular layout, when the location of the target icon changed. This also means that the user interface with circular layout would provide a more stable user experience when the locations of the interface icons change.

## 5. Conclusion

In summary, this study explored more microscopic research issues in interactive interface layout research, such as the influence of the graphic types of target icons, the layout locations of target icons, and the layout methods of target icons on user experience. According to the experimental results of this study, if users do not have cognitive difficulty with each icon in the interactive interfaces, the icon graphic types do not affect the user’s performance or user experience without changing other variables. However, when other variables are changed, such as the icon locations, in general, the design of facet icons will bring more stable user experience than the design of linear icons. Regarding the influence of the layout locations of the target icon on the user experience, it has a lot to do with the layout method of icons in the interactive interface where the target icon is located. For example, in this study, in the circular uniform layout, the influence of the layout locations of the target icon on the user experience was significantly less than that in the rectangular uniform layout.

However, it should be noted that there are limitations for this study. For example, the subjective feeling data of participants were not collected, and only objective eye-movement data were collected to assess the user experience of the interactive interface; the participants involved in the experiment were mainly young women and men, without covering more age groups; the influence of the layout methods on user experience was not obtained by direct comparison, but by indirect way, etc. Further research will focus on addressing these main limitations of this study. The next step would be to introduce the NASA-TLX (Task Load Index) questionnaire into the study to evaluate the users’ subjective feelings from multiple perspectives, including mental demands, physical demands, temporal demands, own performance, effort, and frustration during the completion of the experimental task. At the same time, future research will involve more age groups; more non-uniformly distributed interaction interface layouts; more interaction environments, no longer limited to the automobile console interface, etc., so that the research finds can be adapted to a wider range.

## Data availability statement

The raw data supporting the conclusions of this article will be made available by the authors, without undue reservation.

## Ethics statement

The studies involving human participants were reviewed and approved by Science and Technology Ethics Review Committee of Shanghai Jiao Tong University. The patients/participants provided their written informed consent to participate in this study.

## Author contributions

YZ contributed to conceptualization, methodology, validation, formal analysis, investigation, data curation, writing—original draft, and visualization. JQ contributed to writing—review and editing and project administration. ZF and ZW contributed to conceptualization and methodology. HX, SW, and NZ contributed to validation, investigation, and resources. JH contributed to funding acquisition and writing—review and editing. All authors contributed to the article and approved the submitted version.

## Funding

This research was supported by the National Key Research and Development Program of China (2022YFB3402001), National Natural Science Foundation of China (51975360, 52035007).

## Conflict of interest

The authors declare that the research was conducted in the absence of any commercial or financial relationships that could be construed as a potential conflict of interest.

## Publisher’s note

All claims expressed in this article are solely those of the authors and do not necessarily represent those of their affiliated organizations, or those of the publisher, the editors and the reviewers. Any product that may be evaluated in this article, or claim that may be made by its manufacturer, is not guaranteed or endorsed by the publisher.
